# Highly Porous Type II Collagen-Containing Scaffolds for Enhanced Cartilage Repair with Reduced Hypertrophic Cartilage Formation

**DOI:** 10.3390/bioengineering9060232

**Published:** 2022-05-26

**Authors:** Claudio Intini, Tom Hodgkinson, Sarah M. Casey, John P. Gleeson, Fergal J. O’Brien

**Affiliations:** 1 Tissue Engineering Research Group, Department of Anatomy & Regenerative Medicine, Royal College of Surgeons in Ireland (RCSI) University of Medicine and Health Sciences, D02 YN77 Dublin, Ireland; claudiointini@rcsi.ie (C.I.); tomhodgkinson@rcsi.ie (T.H.); sarahmcasey@rcsi.ie (S.M.C.); john.p.gleeson@dcu.ie (J.P.G.); 2 Advanced Materials and Bioengineering Research (AMBER) Centre, RCSI & Trinity College Dublin (TCD), D02 W085 Dublin, Ireland; 3 Fraunhofer Project Centre for Embedded Bioanalytical Systems, Dublin City University, Glasnevin, D09 Y074 Dublin, Ireland; 4 Trinity Centre for Biomedical Engineering, TCD, D02 R590 Dublin, Ireland

**Keywords:** cartilage repair, biomimetic scaffold, type II collagen, chondrogenesis, hypertrophic cartilage

## Abstract

The ability to regenerate damaged cartilage capable of long-term performance in an active joint remains an unmet clinical challenge in regenerative medicine. Biomimetic scaffold biomaterials have shown some potential to direct effective cartilage-like formation and repair, albeit with limited clinical translation. In this context, type II collagen (CII)-containing scaffolds have been recently developed by our research group and have demonstrated significant chondrogenic capacity using murine cells. However, the ability of these CII-containing scaffolds to support improved longer-lasting cartilage repair with reduced calcified cartilage formation still needs to be assessed in order to elucidate their potential therapeutic benefit to patients. To this end, CII-containing scaffolds in presence or absence of hyaluronic acid (HyA) within a type I collagen (CI) network were manufactured and cultured with human mesenchymal stem cells (MSCs) *in vitro* under chondrogenic conditions for 28 days. Consistent with our previous study in rat cells, the results revealed enhanced cartilage-like formation in the biomimetic scaffolds. In addition, while the variable chondrogenic abilities of human MSCs isolated from different donors were highlighted, protein expression analysis illustrated consistent responses in terms of the deposition of key cartilage extracellular matrix (ECM) components. Specifically, CI/II-HyA scaffolds directed the greatest cell-mediated synthesis and accumulation in the matrices of type II collagen (a principal cartilage ECM component), and reduced deposition of type X collagen (a key protein associated with hypertrophic cartilage formation). Taken together, these results provide further evidence of the capability of these CI/II-HyA scaffolds to direct enhanced and longer-lasting cartilage repair in patients with reduced hypertrophic cartilage formation.

## 1. Introduction

The ability to restore damaged cartilage, providing long-term performance and functional recovery with minimal risk of revision surgery, remains an unmet clinical challenge [1,2]. Surgical intervention is often necessary to treat the majority of articular cartilage defects [3,4]. However, while a number of surgical options are currently available, no conventional approach has demonstrated a capacity to fully restore the damaged cartilage [5,6]. Thus, a clear clinical need for the development of improved treatments for articular cartilage injures, yielding higher-quality repair and longer-term performance in patients with minimal risk of revision surgery remains, and is focus of this study [1,2]. In this context, tissue engineering and regenerative medicine (TERM) offers promising alternative approaches to tackle the treatment of articular cartilage defects and has the potential to fully restore the damaged tissue [7,8]. In particular, biomimetic biomaterials such as collagen-based scaffolds have been shown to be capable of directing effective chondrogenic responses in mesenchymal stem cells (MSCs), resulting in the synthesis of cartilage-like extracellular matrix (ECM) [9,10,11,12]. However, while MSCs have been extensively used with good success in the field, their use has been limited by the tendency of MSCs towards terminal chondrocytic hypertrophic differentiation, subsequently leading to aberrant calcified cartilage and endochondral bone formation [13,14,15]. This is a major barrier in the application of these therapeutic cells, given that the resulting hypertrophic and non-functional cartilage-like matrix is incapable of providing the fundamental biomechanical properties to the engineered tissue [16,17]. Therefore, the development of alternative, more biologically active scaffold biomaterials capable of improving MSC chondrogenesis and consequently enhancing the quality of cartilage-like ECM formed is essential to establish longer-term cartilage repair with improved functional performance in patients.

Accordingly, current research in the field is focusing on the development of more bioactive materials that can provide the correct mechanical and biochemical cues to cells to improve the type and quality of engineered tissue produced [14,15,18,19,20]. Several studies to date have clearly demonstrated that biomaterials can successfully interact with cells via membrane receptors (e.g., integrins) and cell-adhesion attachment motifs activating specific intracellular signalling pathways [21,22,23,24]. Therefore, it is expected that the specific biochemical composition of future biomaterials might play a major role in increasing the quality of engineered tissues, with the ultimate goal to develop longer-term articular-cartilage repair strategies [23,25]. In this scenario, scaffolds mimicking the composition and anatomical arrangement of articular-cartilage ECM have shown some success in modulating MSC hypertrophic differentiation, resulting in the formation of higher quality cartilage-like matrix formation [14,23,26,27]. Specifically, type II collagen (CII)-based biomaterials have demonstrated some potential in reducing cellular hypertrophic events and in enhancing the quality of the newly synthesised cartilaginous matrices [23,24,28,29]. Of special note, a study by Lian C. et al. indicated some promise for CII to suppress late-stage chondrogenesis/hypertrophy and calcified cartilage formation with decreased cell expression of type X collagen (COLX) by human MSCs cultured in a pellet system [23]. Moreover, a recent study in our laboratory has revealed the potential for CII-containing scaffold biomaterials to direct a more controlled MSC differentiation to late-stage chondrogenesis *in vitro* [9]. This study was carried out using rat cells. Building on this knowledge, and with a view towards translation to humans, the research presented in this study has focused on further elucidating the effect of CII on human MSC late-stage chondrogenic differentiation and hypertrophy when incorporated into three-dimensional (3D) type I collagen (CI)-based scaffolds in the presence or absence of hyaluronic acid (HyA). To this end, the specific aims were (1) to investigate the ability of CII containing scaffolds to direct an effective human MSC chondrogenic response and (2) to elucidate their ability to affect human MSC hypertrophic differentiation and the formation of calcified cartilage and endochondral bone.

## 2. Materials and Methods

### 2.1. Fabrication of Collagen-Based Scaffolds

To fabricate the collagen-based scaffolds, a freeze-drying (lyophilisation) method previously described and regularly used in our research group was used [30]. Type I collagen (CI), hyaluronic acid (HyA) and type II collagen (CII) were used at various ratios to prepare a series of CI-based scaffold iterations (Table 1). CI scaffolds were composed of collagen derived from bovine Achilles tendon (Collagen Matrix, Oakland, NJ, USA). CI and HyA (CI-HyA) scaffolds were fabricated using CI and HyA sodium salt derived from *Streptococcus equi* (molecular weight 1500–1800 kDa) (Sigma-Aldrich, Arklow, Ireland). Finally, combination CI/II scaffolds and CI/II-HyA scaffolds were manufactured by incorporating CII isolated from porcine knee cartilage (Symatese, Chaponost, France) into CI and CI-HyA scaffolds, respectively. Briefly, slurries with a total collagen concentration of 0.5% (*w*/*v*) were prepared using both CI and CII at a ratio of 1:1, type I:type II collagen (CI/II) by suspending the collagen in 0.5 M acetic acid as previously described [9]. Similarly, CI/II-HyA slurries were prepared with the same 1:1 type I:type II collagen ratio and final concentration of 0.5% (*w*/*v*) total collagen, in the presence of HyA 0.05% (*w*/*v*). Subsequently, 0.3 mL of each slurry was pipetted into a stainless-steel tray (internal dimensions: 9.5 mm diameter and 4 mm height) before freeze-drying (Virtis Genesis 25EL, Biopharma, Winchester, UK) at a constant cooling rate of 1 °C/min to a final temperature of −20 °C and drying at a pressure of 200mTorr. Following this, the porous scaffolds were dehydrothermally (DHT) crosslinked in a vacuum oven (VacuCell, MMM, Planegg, Germany) for 24 h at a pressure of 0.05 bar and a temperature of 105 °C.

### 2.2. Cell Culture

#### 2.2.1. Bone-Marrow-Derived Mesenchymal Stem Cells in Monolayer

To obtain a sufficient quantity of human bone-marrow MSCs, cells were first expanded in monolayer prior to seeding on the scaffolds. MSCs were either purchased from Lonza (Basel, Switzerland) or isolated from the iliac crest of adults (donor age range: 20–30 years old) using standardised protocols and including a stringent analysis of cell phenotype as previously described [31]. Cells were incubated with low-glucose Dulbecco’s modified Eagle’s medium (DMEM) (Sigma-Aldrich, Arklow, Ireland) supplemented with 10% fetal bovine serum (ThermoFisher Scientific, Dublin, Ireland) and 1% penicillin/streptomycin (ThermoFisher Scientific, Dublin, Ireland). Cells from donors 1, 2 and 3 were passaged using trypsin ethylenediaminetetraacetic acid (EDTA, Sigma-Aldrich, Arklow, Ireland) once confluent, and replated onto T-175 (175 cm^2^ growth area) flasks under normoxic cell-culture conditions (37 °C, 5% CO_2_, 21% O_2_). Cells were cultured on the scaffolds at passage number 4.

#### 2.2.2. Bone-Marrow-Derived Mesenchymal Stem-Cell Seeding on Scaffolds

**Figure 1:**
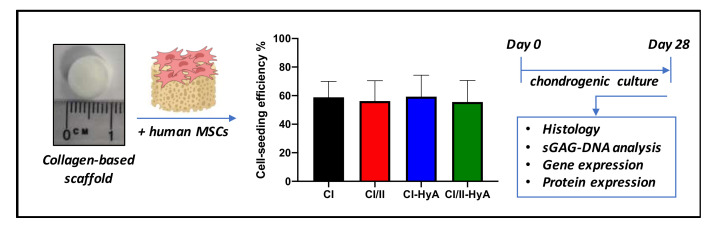
Illustrative scheme describing the experimental design. Collagen-based scaffolds were seeded with human MSCs and processed for histological, sGAG/DNA and gene/protein expression analysis after 28 days in culture.

Human bone-marrow MSCs were detached from their culture flasks via trypsinisation. Cells were counted and resuspended at a density of 5 × 10^5^ cells per scaffold in a total volume of 100 μL. Scaffolds of 9.5 mm diameter and 4 mm height were pre-hydrated in PBS (Sigma-Aldrich, Arklow, Ireland) for 15 min and placed in 24-well plates. The cell suspension was then added to the scaffolds, with 50 μL first pipetted onto one side of each scaffold, before incubating for 30 min (37 °C, 5% CO_2_, 21% O_2_) to allow initial cell attachment. Seeded scaffolds were subsequently turned over and the procedure repeated on the other side. Following this, 2 mL of expansion/growth medium was added to each well and pre-cultured for 24 h. Then, the standard growth medium was replaced with medium supplemented with chondrogenic factors. This chondrogenic media was composed of serum-free high-glucose DMEM supplemented with 50 μg/mL ascorbic acid (Sigma-Aldrich, Arklow, Ireland), 40 μg/mL proline (Sigma-Aldrich, Arklow, Ireland), 100 nM dexamethasone (Sigma-Aldrich, Arklow, Ireland), 1X ITS (BD Biosciences, Wokingham, UK), 0.11 mg/mL sodium pyruvate (Sigma-Aldrich, Arklow, Ireland), and 10 ng/mL human TGF-β3 (Prospec, Rehovot, Israel). Leftover cells attached at the bottom of each well during the scaffold cell-seeding procedure were detached and counted using a haemocytometer. Then, seeding efficiency was calculated by dividing the effective number of cells seeded on the scaffold (total amount of cells seeded per scaffold minus the leftover cells attached at the bottom of the well) to the total amount of cells. The cell-seeded scaffolds were incubated for 28 days, with media being changed twice per week (Figure 1).

### 2.3. Cellular Metabolic-Activity Assay

To determine the metabolic activity of the cells within the scaffolds, an alamarblue assay (ThermoFisher Scientific, Dublin, Ireland) was performed. Scaffolds (*n* = 3 per group) were washed in PBS twice and fresh chondrogenic media containing 10% alamarblue viability reagent were added at 37 °C for 1 h. A spectrophotometer (Victor2 D, Wallac, Boston, MA, USA) with an excitation wavelength of 550 nm and an emission wavelength of 590 nm was used to read the resulting fluorescence level. Chondrogenic media containing 10% alamarblue were used as a blank sample, subtracted from the experimental readings to eliminate background fluorescence. The cellular metabolic activity of cells grown on scaffolds at day 0, 3, 10, 14, 21 and 28 was measured.

### 2.4. DNA Quantification

To assess the DNA content in the scaffolds, a Quant-iT™ PicoGreen^®^ dsDNA assay kit (Invitrogen, Loughborough, UK) was used as per the manufacturer’s instructions. Scaffolds (*n* = 3 per group) were washed in PBS and digested in a papain-enzyme solution prepared with 0.5 M EDTA, cysteine-HCl and 1 mg/mL papain enzyme (Carica papaya, Sigma-Aldrich, Arklow, Ireland) at 60 °C for 12 h. DNA concentration was determined using a standard curve, to give an indication of cell number. The DNA of cells grown on the scaffolds, pretested for cellular metabolic activity, was quantified.

### 2.5. Sulphated Glycosaminoglycan (sGAG) Quantification

To quantify the sulphated glycosaminoglycan (sGAG) content of the scaffolds, a Blyscan sulphated glycosaminoglycan assay kit (Biocolor Life Sciences, Carrickfergus, UK) was used. Scaffolds (*n* = 3 per group) were washed in PBS before digesting in a solution prepared from papain-enzyme solution containing 0.5 M EDTA, cysteine-HCl and 1 mg/mL papain enzyme (Carica papaya, Sigma-Aldrich, Arklow, Ireland) at 60 °C for 12 h. The Blyscan sulphated glycosaminoglycan assay was then performed on the samples as per the manufacturer’s instructions. sGAG concentration was determined using a standard curve.

### 2.6. Histological Analysis of Cellular Infiltration and sGAG Distribution

Histological staining was performed to evaluate cellular infiltration, sGAG distribution and calcium deposition within the scaffolds. Scaffolds were formalin-fixed, paraffin-embedded and transversally sectioned at various depths on a microtome (Leica RM 2255, Leica, Wetzlar, Germany) to give 7 μm-thick sections. These sections were subsequently mounted on Polysine^TM^ glass slides (Fisher-Scientific, Dublin, Ireland), deparaffinised and hydrated before staining. Stains used in the histological analysis were haematoxylin (Sigma-Aldrich, Arklow, Ireland), which stains the DNA and RNA rich cell nuclei blue; eosin (Sigma-Aldrich, Arklow, Ireland), which stains the ECM pink; thionine (Sigma-Aldrich, Arklow, Ireland), which stains sGAG purple; and alizarin red (Sigma-Aldrich, Arklow, Ireland), which stains calcium red [32,33]. The sections were successively imaged at several different magnifications using a Leica DMIL microscope (Leica, Wetzlar, Germany).

### 2.7. Gene Expression Analysis

To determine the expression of specific genetic markers associated with chondrogenic lineage, quantitative reverse transcriptase polymerase chain reaction (qRT-PCR) was conducted (Table 2). The total RNA was isolated from the scaffolds using a RNeasy kit (Qiagen, Manchester, UK) as previously described [34]. RNA was reverse-transcribed to cDNA at a final concentration of 2.5 ng/µL using a QuantiTect reverse-transcription kit (Qiagen, Manchester, UK) on a thermal cycler (Mastercycler Personal, Eppendorf, UK). qRT-PCR reactions were run on a 7500 real-time PCR System (Applied Biosystems, Loughborough, UK) using a QuantiTect SYBR Green PCR Kit (Qiagen, Manchester, UK). The relative expression of mRNA was calculated by delta Ct (∆Ct) method, where delta Ct (∆Ct) is the value obtained by subtracting the cycle threshold value (Ct) of a house-keeping gene, from the Ct value of the target mRNA type II collagen alpha 1 chain (*COL2A1*), type I collagen alpha 1 chain (*COL1A1*), aggrecan (*ACAN*), SRY-Box Transcription Factor 9 (*SOX9*), runt-related transcription factor 2 (*RUNX2*) and type X collagen alpha 1 chain (*COL10A1*). 18S ribosomal RNA (*18S*) was used as the housekeeping gene.

### 2.8. Protein Expression Analysis

To determine the level of expression of specific protein markers of chondrogenic lineage differentiation, Western blotting analysis was conducted. Cell-seeded scaffolds were manually homogenised, before measuring total protein content using a Pierce^TM^ BCA Protein Assay Kit (ThermoFisher Scientific, Dublin, Ireland). 10 μg total protein was incubated at 95 °C for 5 min with loading buffer, before loading into a gradient gel (Bolt 4–12% Bis-Tris Plus, ThermoFisher Scientific, Dublin, Ireland). Proteins were separated by electrophoresis and subsequently transferred to a polyvinylidene fluoride or polyvinylidene difluoride (PVDF) membrane, blocking with 5% *w*/*v* fat-free milk in 0.1% *v*/*v* Tris-buffered saline (TBS)-Tween 20 (TBST) (Sigma-Aldrich, Arklow, Ireland). The membrane was incubated at 4 °C overnight with a 1:1000 solution of diluted rabbit monoclonal [EPR12268] anti-type II collagen (COLII) antibody (Abcam, ab188570), 1:1000 diluted rabbit polyclonal anti-type X collagen (COLX) antibody (Abcam, ab58632) and 1:5000 rabbit polyclonal anti-GAPDH (GAPDH) antibody (Abcam, ab9485). Membranes were washed three times in TBST and then incubated with the respective secondary antibody for 1 h at room temperature (1:5000). Protein bands were visualised using a Super Signal West detection kit (Thermo Fisher Scientific, Dublin, Ireland). Protein bands were quantified using ImageJ2 software version 2.3.0.

### 2.9. Statistical Analysis

Data shown represent the grand mean from three individual human MSC donors (*n* = 3). Statistics were carried out using GraphPad Prism software version 9.2.0 using a general linear-model analysis of uncorrected Fisher’s LSD with individual variances computed for each comparison. *p*-values less than or equal to 0.05 (*p* ≤ 0.05) were considered statistically significant. * denotes *p* ≤ 0.05, ** = *p* ≤ 0.01, *** = *p* ≤ 0.001 and **** = *p* ≤ 0.0001.

## 3. Results

### 3.1. All Scaffolds Sustained Human MSC Viability up to 28 Days in Culture

**Figure 2:**
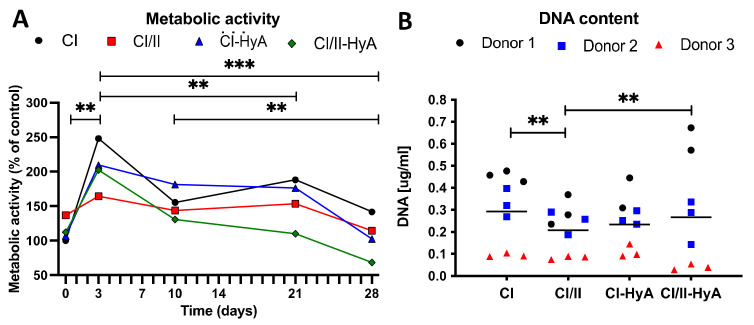
All collagen scaffolds supported human MSC viability up to 28 days in culture. Cellular metabolic activity per scaffold was determined and normalised to CI scaffolds at time 0 after 0, 3, 10, 14, 21 and 28 days in culture (**A**). The DNA content per scaffold was determined after 28 days in culture (**B**). Data shown represent the grand mean from three individual human MSC donors *n* = 3 (unless indicated differently). ** denotes *p* ≤ 0.01 and *** = *p* ≤ 0.001.

To assess biocompatibility, the capacity of the scaffolds to support cellular viability and sustain growth in culture was investigated. Biochemical assays to assess cellular metabolic activity and DNA content revealed that all scaffold variants performed well, demonstrating an equal ability to sustain cellular viability *in vitro* up to day 28 (Figure 2). Specifically, all scaffold variants resulted in significantly increased cellular metabolic activity at day 3 compared to day 0 (*p* ≤ 0.01), 21 (*p* ≤ 0.01) and 28 (*p* ≤ 0.001) (Figure 2A). The composite CI/II scaffolds possessed significantly lower levels of DNA compared to CI (*p* ≤ 0.01) and CI/II-HyA (*p* ≤ 0.01) scaffolds, although there were no statistical differences in DNA content between the other groups (Figure 2B). Scaffolds seeded with cells from donor 1 displayed the highest levels of DNA overall (0.3–0.6 μg/mL) compared to scaffolds seeded with donor 2 (0.2–0.3 μg/mL) and donor 3 (0.05–0.15 μg/mL).

### 3.2. All Scaffolds Supported Effective Deposition of sGAG, with Improved Cartilage-Like Matrix Formation for the Composite CI-HyA and CI/II-HyA Scaffolds

**Figure 3:**
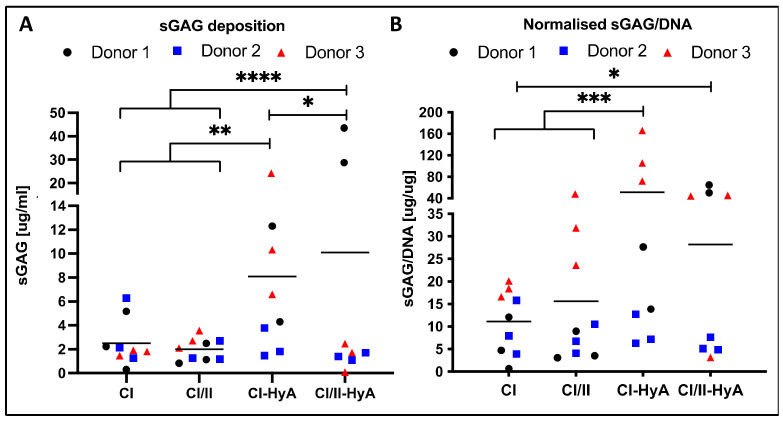
All scaffolds supported effective sGAG deposition by the human MSCs, with improved cartilage-like matrix deposition for the composite CI-HyA and CI/II-HyA scaffolds. Overall sGAG per scaffold (**A**) and sGAG normalised to DNA content (**B**), respectively, were determined after 28 days in culture. Data shown represent the grand mean from three individual human MSC donors *n* = 3 (unless indicated differently). * denotes *p* ≤ 0.05, ** = *p* ≤ 0.01, *** = *p* ≤ 0.001 and **** = *p* ≤ 0.0001.

The ability of the scaffolds to direct MSC chondrogenesis and cartilage-like matrix formation *in vitro* was then evaluated. All scaffold variants sustained sGAG deposition by the cells in the matrices at day 28 (Figure 3). The composite CI/II-HyA scaffolds displayed the highest level of sGAG per scaffold (10.09 μg/mL) (Figure 3A). This was significantly greater than CI (*p* ≤ 0.0001), CI/II (*p* ≤ 0.0001) and CI-HyA (*p* ≤ 0.05) scaffolds. When normalised to DNA, sGAG were higher in CI-HyA (51.46 μg/μg) scaffolds compared to the other groups, with significance to CI (11.13 μg/μg) and CI/II (15.59 μg/μg) scaffolds (*p* ≤ 0.001) (Figure 3B). Although CI/II-HyA (28.15 μg/μg) scaffolds revealed lower sGAG/DNA levels compared to CI-HyA, they displayed significantly higher sGAG/DNA levels than CI (*p* ≤ 0.001) group.

### 3.3. Type II Collagen Incorporation in CI-HyA Scaffolds Enhanced sGAG Distribution without Promoting Further Calcium Deposition

**Figure 4:**
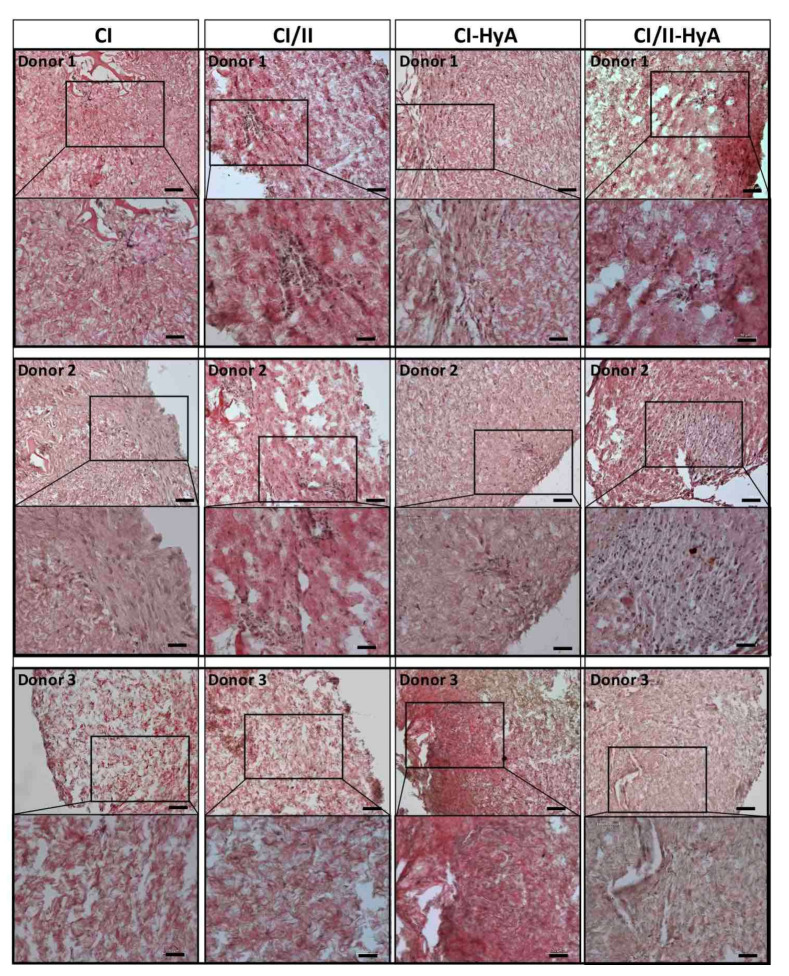
All collagen scaffolds demonstrated an equal capacity to sustain cellular infiltration and migration. Representative histological images of cell-seeded scaffolds stained with haematoxylin and eosin after 28 days in culture. Scale bar represents 100 µm length (**top** images) and 50 µm length (**bottom** images). Histological images were collected for scaffolds seeded with human MSCs from donor 1, 2 and 3, respectively.

**Figure 5:**
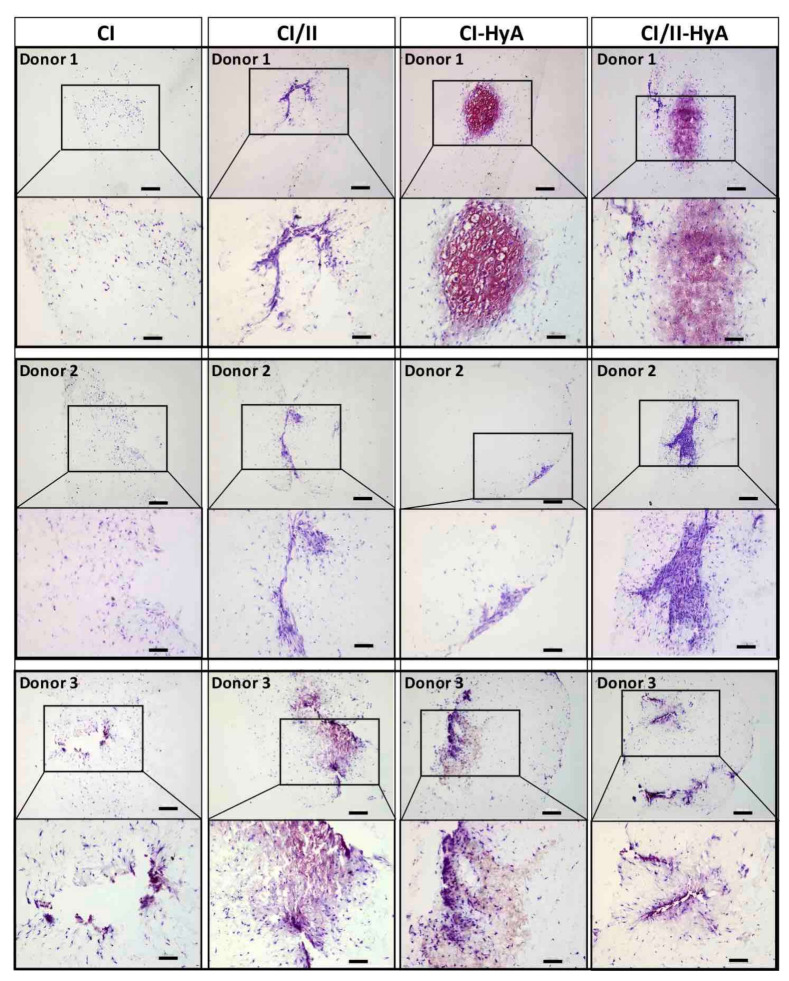
The incorporation of CII in combination with HyA improved sGAG distribution. Representative histological images of cell-seeded scaffolds stained with thionine after 28 days in culture. Scale bar represents 200 µm length (**top** images) and 100 µm length (**bottom** images). The histological images were collected for scaffolds seeded with human MSCs from donors 1, 2 and 3.

**Figure 6:**
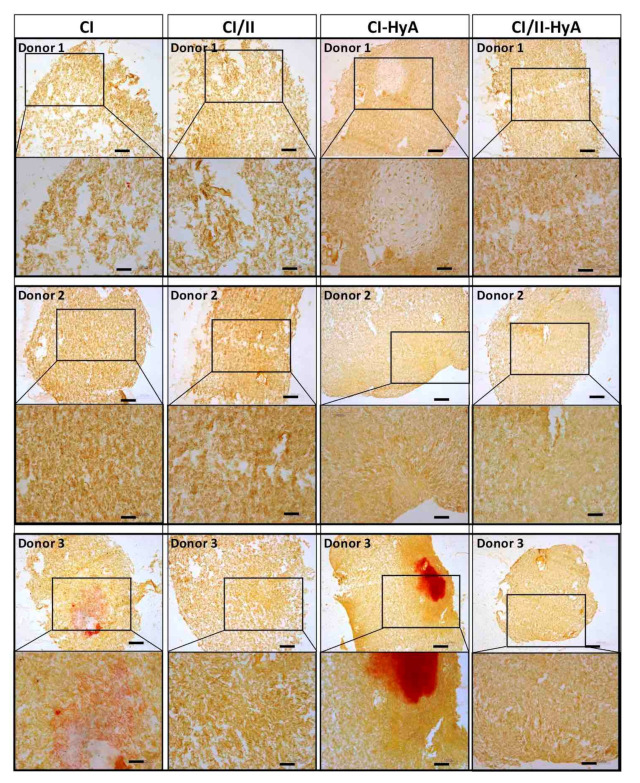
The incorporation of CII did not promote further bone-like tissue formation. Representative histological images of cell-seeded scaffolds stained with alizarin red after 28 days in culture. Scale bar represents 200 µm length (**top** images) and 100 µm length (**bottom** images). The histological images were collected for scaffolds seeded with human MSCs from donors 1, 2 and 3.

To qualitatively assess the ability of the scaffolds to support human MSC migration and cartilage-like matrix distribution (represented by sGAG deposition) in the absence of bone-like matrix in the scaffolds, histological staining of the scaffolds was performed and analysed. Histological analysis revealed that all scaffold variants performed well, with an equal capacity to direct efficacious cellular infiltration and migration throughout the matrices (Figure 4). However, the incorporation of CII in combination with HyA, allowed CI/II-HyA scaffolds to achieve more homogeneous sGAG distribution throughout the matrix compared to the other scaffold variants as shown by thionine staining (Figure 5). Specifically, in the composite CI/II-HyA scaffolds, the majority of sGAG was found in the centre of the scaffold, in contrast to CI/II and CI-HyA scaffolds which primarily demonstrated peripheral distributions of sGAG. Moreover, although CI scaffolds were populated by cells, they generally showed minimal levels of sGAG in the scaffolds. In this context, alizarin red staining revealed that the presence of CII biomaterial allowed the scaffolds to maintain comparably low levels of calcium deposition than scaffolds in the absence of CII (Figure 6). However, although the incorporation of CII did not show reduced levels of calcium deposition with donor 1 and 2, scaffolds in the presence of CII revealed decreased bone-like tissue deposition than CI and CI-HyA scaffolds with donor 3. Overall, the composite CI/II-HyA scaffold induced efficacious cellular migration in parallel to an improved early cartilaginous matrix distribution throughout the scaffolds without promoting further bone-like tissue formation.

### 3.4. All Scaffolds Supported Effective Human MSC Chondrogenic Differentiation, with Reduced Expression of COL10A1 Gene in Composite CI/II-HyA Scaffolds

**Figure 7:**
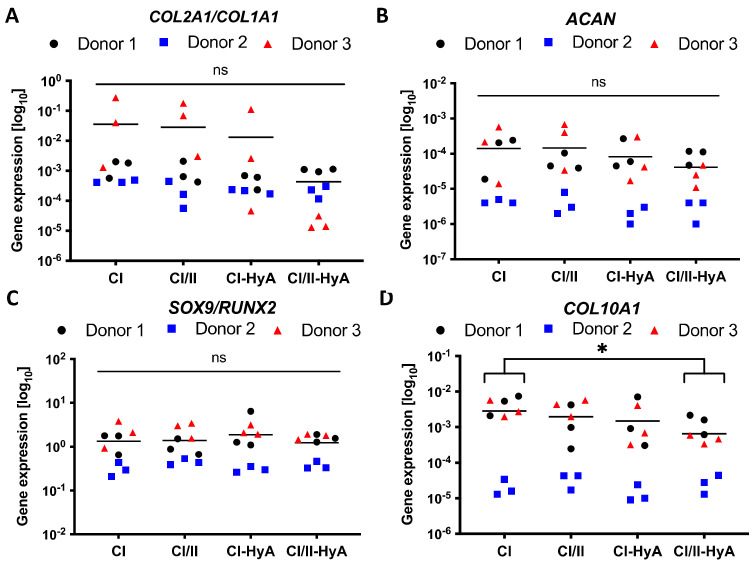
All collagen scaffolds supported the expression of genes associated with effective human MSC chondrogenic differentiation, with reduced expression of *COL10A1* gene in composite CI/II-HyA scaffolds. The expression of *ACAN* (**B**) and *COL10A1* (**D**) genes was determined after 28 days in culture, normalised to *18S* and then converted to relative gene expression using the formula: Fold increase = 2^(−∆Ct). The ratios between *COL2A1*/*COL1A1* and *SOX9*/*RUNX2* genes are shown in (**A**,**C**). Data shown represent the grand mean from three individual human MSC donors *n* = 3. * denotes *p* ≤ 0.05.

The capability of the scaffolds to sustain human MSC chondrogenic differentiation was then investigated by measuring the expression of specific genetic markers typically associated with effective chondrogenesis and late-stage MSC differentiation [35,36]. All scaffold variants sustained *COL2A1*, *COL1A1, ACAN*, *SOX9*, *RUNX2* and *COL10A1* gene expression by the MSCs at day 28 (Figure 7). *COL10A1* gene expression levels were significantly downregulated in the composite CI/II-HyA scaffolds compared to CI (*p* ≤ 0.05), thus indicating the ability of the composite CI/II-HyA scaffolds to reduce hypertrophy, by preventing MSC hypertrophic differentiation and calcified cartilage formation (Figure 7D). There was no statistical difference in *COL2A1*/*COL1A1*, *ACAN* and *SOX9*/*RUNX2* gene expression between the groups (Figure 7A–C).

### 3.5. Type II Collagen Incorporation in CI-HyA Scaffolds Decreased the Synthesis of COLX and Increased the Accumulation of COLII in the Matrices

**Figure 8:**
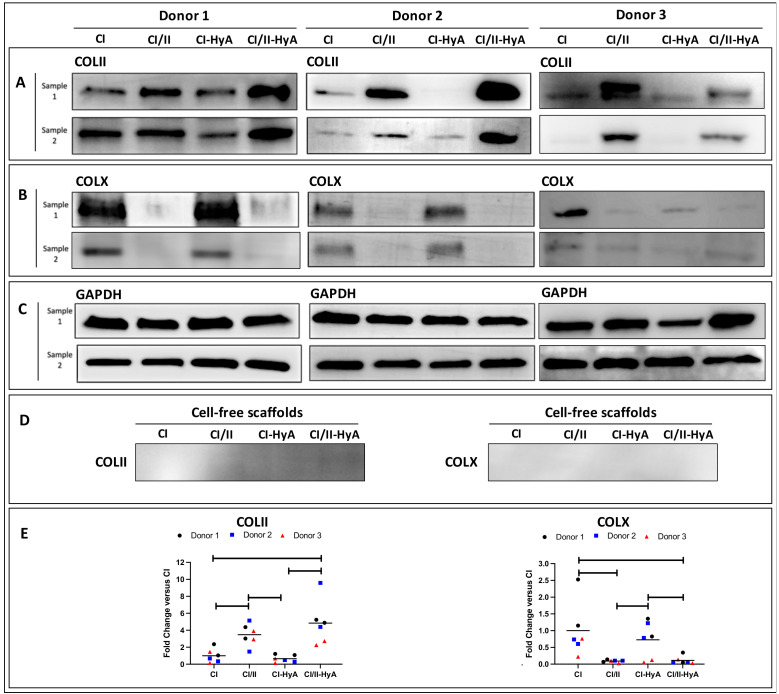
The incorporation of CII was associated with a reduction of COLX synthesis, and in combination with HyA, was found to increase the accumulation of COLII in the matrices by day 28. The expression of COLII (**A**), COLX (**B**) and GAPDH (housekeeper) (**C**) proteins was determined for scaffolds seeded with human MSCs from donors 1, donor 2 and donor 3 after 28 days in culture. COLII and COLX protein expression was also determined on cell-free scaffolds (**D**). The relative protein-band intensity of COLII and COLX was quantified and normalised to GAPDH (**E**).

Having confirmed that the scaffolds were a suitable platform to support MSC chondrogenesis, the expression of key cartilage ECM proteins typically associated with high-quality cartilage-like matrix formation and MSC hypertrophic differentiation was investigated [36,37]. Protein expression analysis revealed that the incorporation of CII in combination with HyA allowed CI/II-HyA scaffolds to accumulate the highest quantities of type II collagen (COLII) protein in the matrices compared to the other groups at day 28 with donor 1 and 2 (Figure 8A,E). Additionally, the synthesis of type X collagen (COLX) protein per scaffold was considerably downregulated by day 28 in the presence of CII biomaterial with donor 1 and 2 (Figure 8B,E). With donor 3, although the incorporation of CII in combination with HyA did not show the highest levels of COLII deposition, CI/II-HyA scaffolds accumulated increased COLII protein than CI and CI-HyA scaffolds. Moreover, low levels of COLX protein were detected in all groups seeded with donor 3 (Figure 8B).

## 4. Discussion

The overall aim of this study was to investigate the ability of type II collagen (CII)-containing scaffolds designed for enhanced cartilage repair to direct an effective human MSC chondrogenic response with reduced formation of calcified cartilage and endochondral bone. To this end, previously designed CII containing scaffolds in presence or absence of hyaluronic acid (HyA) within a type I collagen (CI) network were manufactured and cultured with MSCs from three individual human donors *in vitro* under chondrogenic conditions for 28 days [9]. The results reflected our previous study, which revealed the capacity of the composite CI/II-HyA scaffolds to enhance rat MSC chondrogenesis and early cartilaginous matrix deposition [9], albeit in this study using human primary cells. In addition, the results highlighted the ability of the CI/II-HyA scaffolds to direct reduced formation of hypertrophic cartilage. Specifically, the composite CI/II-HyA scaffolds led to improved human MSC chondrogenesis with the greatest cell-mediated synthesis and accumulation in the matrices of type II collagen (a principal cartilage ECM component), and reduced deposition of type X collagen (a key protein associated with MSC hypertrophic differentiation and calcified cartilage formation) [33,37]. Taken together, this study demonstrates the ability of CI/II-HyA scaffolds to enhance human MSC chondrogenesis while repressing further differentiation toward endochondral bone formation, thus providing promise for enhanced cartilage repair, with the potential of longer-lasting treatments in patients with articular joint injuries.

Complete analysis revealed that biologically, all collagen-based scaffolds performed well, supporting effective human MSC viability, migration and early cartilaginous-matrix deposition throughout the scaffolds. These results are not unexpected and reflect what was observed previously in our laboratory and in other studies evaluating the capacity of highly porous interconnected collagen-based scaffolds to facilitate efficacious cellular viability and matrix formation [11,12,38,39]. Moreover, the incorporation of CII in combination with HyA in CI/II-HyA scaffolds has been shown to improve early cartilaginous-matrix deposition and distribution (represented by sGAG) in the matrices as previously shown in our laboratory [9]. Therefore, the results demonstrate and confirm the importance of these cartilage ECM components to play a synergistic role in directing enhanced human MSC chondrogenesis and cartilage-like ECM formation *in vitro*. In addition, building on this earlier work, the results successfully translated to a human MSC-based study that represents the ideal choice of model to assess a scaffold biomaterial prior to further investigation *in vivo* and translation for human applications [40,41].

Another significant beneficial effect observed when combining CII with HyA was a more robust and higher-quality MSC-mediated chondrogenic response, which translated to increased deposition of COLII in the scaffolds at day 28. COLII is a major cartilage ECM protein composing the fibrillar collagen network in articular cartilage, which in combination with HyA and other minor components provides a supramolecular structural organisation that is essential to maintain relevant mechanical properties and provide integrity to the whole tissue [22,37]. With this in mind, the accumulation of COLII in the newly synthesised cartilage-like matrices in cartilage TERM remains one of the essential achievements that indicates increased quality of the engineered matrices [36,37]. In this study, protein expression analysis revealed that the composite CI/II-HyA scaffolds accumulated the highest levels of COLII at day 28 compared to the other scaffold variants. This specific beneficial effect of improved COLII deposition observed when combining CII with HyA in 3D porous scaffolds has not previously been described in the literature. However, the results were supported by a similar study showing that the combination of CII with chondroitin sulphate (another articular cartilage ECM component) in 3D scaffolds is critical to achieve increased COLII deposition by adipose-derived MSCs at day 28 [42]. Therefore, as discussed earlier, these findings suggest that the combination of CII with HyA is essential to enhance MSC chondrogenesis and early cartilaginous matrix deposition.

Protein expression analyses also revealed the promising ability for the composite CI/II-HyA scaffold to reduce hypertrophy by preventing MSC hypertrophic differentiation and calcified cartilage formation. In particular, the incorporation of CII into the scaffold led to improved MSC chondrogenesis with reduced cell-mediated deposition of COLX (a key protein associated with MSC hypertrophic differentiation) by day 28 [15,37]. Interestingly, the results suggested a potential independent role played by CII in controlling cellular hypertrophic events with human MSCs, consequently promoting higher quality engineered cartilage-like matrices. Previous studies have shown some potential for CII to direct effective MSC chondrogenic differentiation while reducing MSC’s hypertrophic tendency towards endochondral bone formation [23,24,28,29]. The results in this study were similar to those of a recent study showing a significant influence of CII on human MSCs that were firstly induced to chondrogenesis, and then to hypertrophy, downregulating COLX protein expression by the cells at day 28 [23]. In this study by Lian C. *et al*., CII’s ability to direct decreased expression of COLX through activation of the ERK1/2−SMAD1 pathway was proven, with consequent bone morphogenetic protein (BMP) inhibition [23]. Therefore, it is possible to speculate that the presence of CII in collagen-based scaffolds plays a major role as a signalling molecule that upon interaction with integrin β1 receptors activates the ERK1/2 complex, which in turn phosphorylates SMAD1, causing its inhibition. This provides some promise for CII to repress MSC hypertrophic differentiation toward endochondral bone formation. Further research could be undertaken to gain a deeper understanding of the cellular and molecular mechanisms governing this possible effect of CII when incorporated into 3D collagen-based biomaterials.

A full donor-by-donor analysis highlighted variable abilities of the human MSCs isolated from different individuals (donors 1, 2 and 3) to proliferate and respond to the chondrogenic stimuli provided in the scaffolds. This MSC heterogenicity is not a new phenomenon and is well-established in the literature, with several other studies revealing recurrent MSC donor-to-donor variability in various cell responses [43,44,45,46]. Consequent to our findings, the level of complexity in understanding specific donor-to-donor MSC responses has increased, and we believe this is an important factor which needs further evaluation and deeper comprehension in the field. In this study, scaffolds seeded with donor 3 presented the lowest overall levels of DNA, with peak cellular metabolic activity delayed by approximately 10 days compared to scaffolds seeded with donors 1 or 2 (data not shown). It is known that differentiation is a metabolically expensive cellular process that implicates a metabolic shift and higher levels of metabolism compared to cells during proliferation and expansion [47,48]. It can thus be assumed that the MSCs from donor 3 were initially less responsive to chondrogenesis and the chondrogenic cues, thus resulting in reduced differentiation and proliferation compared to donors 1 and 2. This may explain why low levels of DNA and overall sGAG were observed in the scaffolds seeded with donor 3. Moreover, low levels of COLX in parallel with increased calcium-like matrix deposition in scaffolds in the absence of CII were observed with donor 3. These findings support our initial hypothesis on CII effect in controlling cellular hypertrophic events and possibly suggest a more osteogenic-like behaviour of the MSCs isolated from donor 3, resulting in the formation of endochondral bone-like tissue. In terms of cartilage-like matrix deposition, donor 1 promoted the highest levels of sGAG compared to donors 2 and 3, which might highlight a better response to chondrogenesis in this group. Nevertheless, in the same context, donor 2 was associated with the lowest levels of gene expression and sGAG deposition. Unlike donor 3, which demonstrated a stronger osteogenic response (as previously discussed), the data suggest that donor 2 possessed intrinsically low chondrogenic/osteogenic ability with minimal capacity to respond to chondrogens. Therefore, while these findings highlight the donor-to-donor variability of these human MSCs, particularly in relation to sGAG levels between donors, protein expression analysis illustrated consistent responses in terms of the deposition of key cartilage ECM qualitative components such as COLII and COLX by the cells. We suggest that this increases the significance and impact of these findings and demonstrates the ability of CI/II-HyA scaffolds in directing enhanced, higher quality, cartilage-like matrix formation with reduced calcified cartilage formation. Taken together, the composite CI/II-HyA scaffolds have provided *in vitro* evidence of their promise in acting as a prospective “off-the-shelf” therapeutic biomaterial for enhanced and longer-lasting cartilage repair; further investigation *in vivo* is merited.

## 5. Conclusions

The results from this study demonstrate the ability of a highly porous type II collagen-containing scaffold, in combination with hyaluronic acid and type I collagen, to direct more effective human MSC chondrogenesis with reduced hypertrophy and calcified cartilage-like formation after 28 days of culture. This study provides evidence that the CI/II-HyA scaffold is a promising biomaterial warranting further investigation *in vivo*, with strong potential for future use as a simple and prospective “off the shelf” therapeutic approach to support longer-lasting cartilage-repair strategies in the clinic.

## Figures and Tables

**Table 1:** Experimental collagen-based scaffolds manufactured and investigated for cartilage-like tissue regeneration. Scaffold biomaterials composed of type I collagen (CI) and type II collagen (CII) at various ratios were prepared to a final collagen concentration of 0.5% (*w*/*v*), in the presence or absence of hyaluronic acid (HyA) at concentration of 0.05% (*w*/*v*).

Scaffold type	CI% (*w/v*)	HyA% (*w/v*)	CII% (*w/v*)
CI	0.50%	/	/
CI/II	0.25%	/	0.25%
CI-HyA	0.50%	0.05%	/
CI/II-HyA	0.25%	0.05%	0.25%

**Table 2:** List of gene transcripts analysed by qRT-PCR. Qiagen QuantiTect validated primers used to analyse the expression levels of target genes.

Target Gene	Target Gene Referencce	Catalogue Code
Collagen type II alpha 1 chain (*COL2A1*)	Hs_COL2A1_1_SG	QT00049518
Collagen type I alpha 1 chain (*COL1A1*)	Hs_COL1A1_1_SG	QT00037793
Aggrecan (*ACAN*)	Hs_ACAN_1_SG	QT00001365
SRY-Box Transcription Factor 9 (*SOX9*)	Hs_SOX9_1_SG	QT00001498
Runt-related transcription factor 2 (*RUNX2*)	Hs_RUNX2_1_SG	QT00020517
Collagen type X alpha 1 chain (*COL10A1*)	Hs_COL10A1_1_SG	QT00096348
18s ribosomal RNA (*18S*)	Hs_RRN18S_1_SG	QT00199367

## Data Availability

Not applicable.
